# Statins Disrupt CCR5 and RANTES Expression Levels in CD4^+^ T Lymphocytes In Vitro and Preferentially Decrease Infection of R5 Versus X4 HIV-1

**DOI:** 10.1371/journal.pone.0000470

**Published:** 2007-05-23

**Authors:** Alexey A. Nabatov, Georgios Pollakis, Thomas Linnemann, William A. Paxton, Michel P. de Baar

**Affiliations:** 1 Laboratory of Experimental Virology, Department of Medical Microbiology, Center of Infection and Immunity Amsterdam (CINIMA), Academic Medical Center of the University of Amsterdam, Amsterdam, The Netherlands; 2 Primagen, Amsterdam, The Netherlands; University of California, San Francisco, United States of America

## Abstract

**Background:**

Statins have previously been shown to reduce the in vitro infection of human immunodeficiency virus type 1 (HIV-1) through modulation of Rho GTPase activity and lipid raft formation at the cell surface, as well as by disrupting LFA-1 incorporation into viral particles.

**Principle Findings:**

Here we demonstrate that treatment of an enriched CD4^+^ lymphocyte population with lovastatin (Lov), mevastatin (Mev) and simvastatin (activated and non-activated, Sim(A) and Sim(N), respectively) can reduce the cell surface expression of the CC-chemokine receptor CCR5 (P<0.01 for Sim(A) and Lov). The lowered CCR5 expression was associated with down-regulation of CCR5 mRNA expression. The CC-chemokine RANTES protein and mRNA expression levels were slightly increased in CD4^+^ enriched lymphocytes treated with statins. Both R5 and X4 HIV-1 were reduced for their infection of statin-treated cells; however, in cultures where statins were removed and where a decrease in CCR5 expression was observed, there was a preferential inhibition of infection with an R5 versus X4 virus.

**Conclusions:**

The results indicate that the modulation of CC-chemokine receptor (CCR5) and CC-chemokine (RANTES) expression levels should be considered as contributing to the anti-viral effects of statins, preferentially inhibiting R5 viruses. This observation, in combination with the immunomodulatory activity exerted by statins, suggests they may possess more potent anti-HIV-1 activity when applied during the early stages of infection or in lowering viral transmission. Alternatively, statin treatment could be considered as a way to modulate immune induction such as during vaccination protocols.

## Introduction

Antiretroviral therapy has expanded the lives of many infected with HIV-1, however, emerging resistance and the encountered toxicity indicate that new classes of drugs capable of reducing virus replication are desired. In clinical trials the cholesterol lowering class of drugs termed statins have been shown to be beneficial in the primary and secondary prevention of coronary heart disease [1]. Recently a number of statins have been shown to possess anti-HIV-1 activity through a number of mechanisms and have been proposed for the treatment of HIV-1 infection. Two main mechanisms have been proposed to contribute to this inhibitory effect; 1) the down-modulation of lipid raft formation through modulation of Rho GTPase activity and 2) the blocking of the interaction between virion-associated ICAM-1 and cell associated LFA-1 [2], [3]. Statins strongly inhibit the endogenous cholesterol biosynthesis by inhibiting the rate-limiting enzyme in this biosynthesis process, named [3-hydroxy-3-methylglutaryl coenzyme A (HMG-CoA) reductase]. Inhibition of small GTP-binding proteins, Rho, Ras, and Rac, whose proper membrane localization and function are dependent on isoprenylation, are supposed to be responsible for the pleiotropic effects of statins [4], [5]. Several *in vivo* studies have suggested a beneficial effect of statins on HIV-1 viral load measurements, which are highly predictive for disease progression, whilst others have reported no beneficial effect of statins in controlled trials [2], [6], [7].

The chemokine receptors CCR5 and CXCR4 have been shown to serve, in conjunction with the primary CD4 receptor, as HIV-1 coreceptors, which are required for viral entry [8]–[10]. The classification of HIV tropism is based on chemokine receptor usage of either CCR5 (R5 virus), CXCR4 (X4 virus), or both receptors (R5X4 virus), although the utilization of other chemokine receptors has been reported [11], [12]. These receptors mediate immune cell responses to a family of soluble chemo-attractant molecules, termed chemokines. The CC chemokines RANTES, MIP-1α and MIP-1β, the natural ligands for the CCR5 chemokine receptor, and SDF-1α, the natural ligand for the CXCR4 coreceptor, have been shown to successfully block the replication of HIV-1 in vitro [13], [14]. These receptors are therefore likely targets for drug development provided that no essential cellular functions are affected by the intervention strategy. Interestingly, it has previously been reported that statins can down-regulate the levels of the CCR5 chemokine receptor on both B and T lymphocytes [15]

CCR5 has been shown to be present in cholesterol rich lipid rafts, co-localizing at the leading edge of migrating cells [16]. This receptor, in contrast to CXCR4, has also been shown to be palmitoylated, which is one of the important modifications in lipid raft targeting of proteins [17]–[19]. Importance of the cholesterol presence in the membrane has recently been demonstrated in experiments studying the effects of cyclodextrins on CCR5 and CXCR4 function as both chemokine receptors and as HIV-1 co-receptors [20], [21].

## Results and Discussion

### Statins down-modulate expression of CCR5 on CD4^+^ T lymphocytes

**Figure 1:**
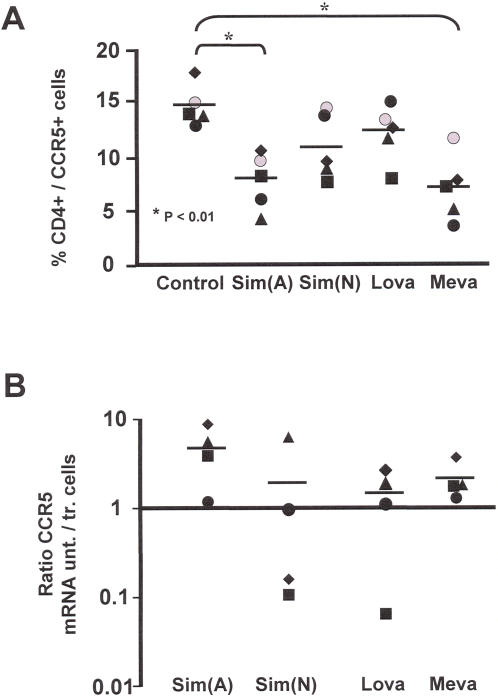
Statins down-regulate expression of CCR5 on CD4^+^ T lymphocytes. (A) CCR5 cell surface expression was monitored on enriched CD4^+^ T lymphocytes treated with Lov, Mev, Sim(A) and Sim(N). Mean levels of CCR5 expression in the control and statin treated cells are depict, * indicates P<0.01. (B) Levels of CCR5 mRNA were assayed in CD4^+^ T lymphocytes treated with Lov, Mev, Sim(A) and Sim(N) on day 4 of treatment. The results are presented as a ratio between untreated (unt.) cells and cells treated (tr.) with statins.

We aimed to analyse whether statins affected CCR5 cell surface expression on CD4^+^ T lymphocytes isolated from 5 separate individuals. Isolated CD4^+^ T lymphocytes were activated and treated with either Sim(N), Sim(A), Lov or Mev and compared for CCR5 expression on day 3 of treatment. Untreated cells from the same donors were used as controls (Figure 1A). A final concentration of 5 µM was utilized for all statins tested with no alterations to either CD4^+^ T lymphocyte cell counts or cell viabilities identified (data not shown), indicating lack of toxicity induced by statins. We found that all statins induced a reduction in the cell surface expression of CCR5 (Figure 1A). There was a statistical significant reduction in CCR5 expression for Sim(A) and Mev treatment (P<0.01) with a trend towards a reduced expression with Sim(N) and Lov. The lower reduction in CCR5 expression observed with Sim(N) likely reflects the lower activity of this drug over its activated form and suggests that the activity of the compound is responsible for the lowered expression of CCR5. We tested the expression of CD4 and CXCR4 expression following statin treatment and although a slight reduction in CD4 expression was observed no decrease in CXCR4 expression was identified for any of the statins tested (data not shown). This latter finding indicates that the lowered CCR5 expression is specific and not due to toxicity.

We proceeded to determine whether the effect of statins on lowering CCR5 cell surface expression was due to a reduction in CCR5 mRNA expression or alterations to protein trafficking and CCR5 association with the cell membrane. We developed and utilized a Nucelic Acid Sequence Based Amplification (NASBA) mRNA quantitative assay and utilized it to measure CCR5 mRNA levels in CD4^+^ T lymphocytes. Overall a slight, but not significant, reduction in CCR5 mRNA expression was detected when comparing untreated versus statin treated cells (Figure 1B), with Sim(A) showing the lowest reduction in mRNA synthesis. The results indicate that the reduced cell surface expression of CCR5 on CD4^+^ T lymphocytes can only in part be explained by down-regulation of mRNA expression.

### Statins up-regulate the expression of RANTES in CD4^+^ T lymphocytes

**Figure 2:**
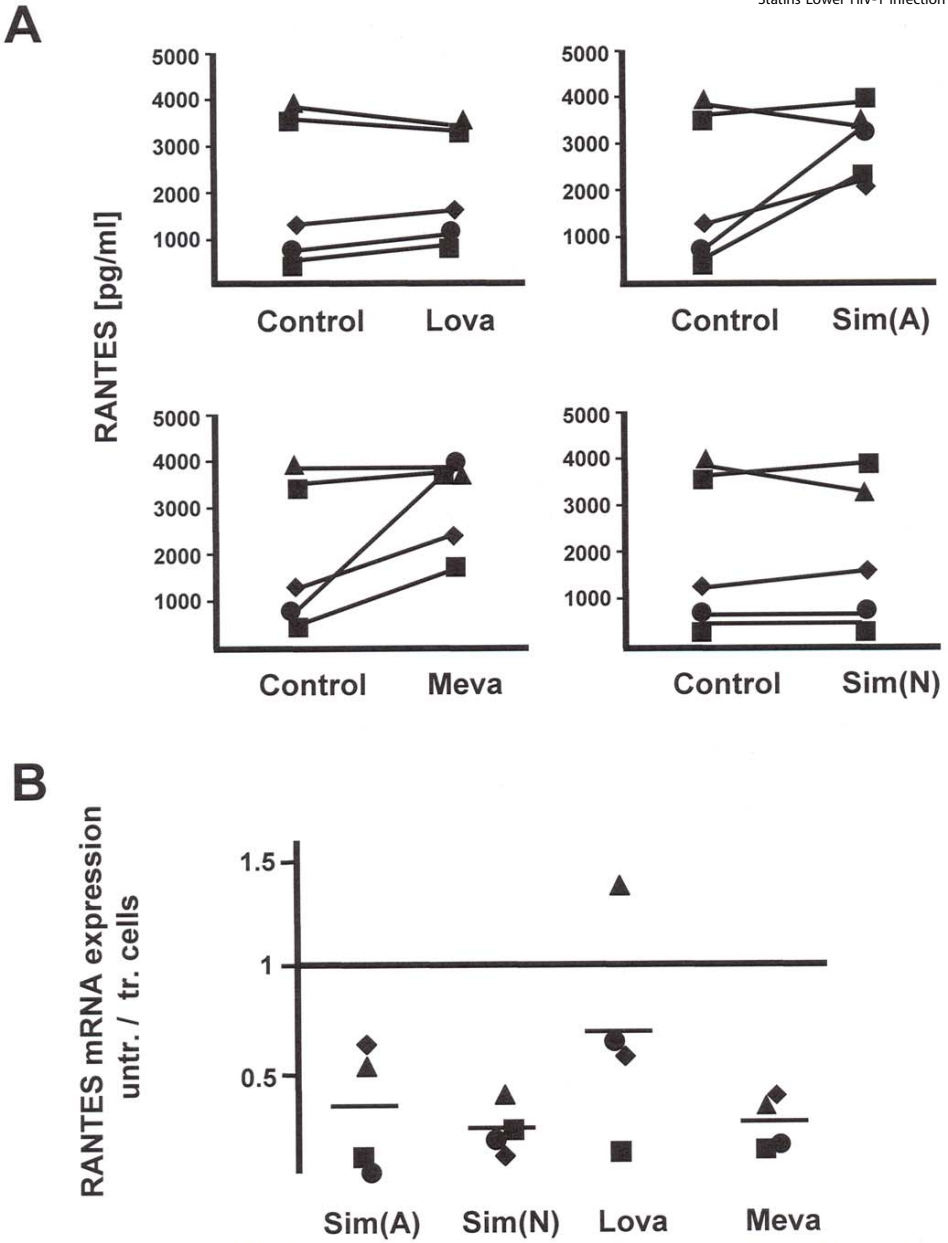
Statins upregulate RANTES accumulation in culture supernatant and increase RANTES mRNA expression. (A) RANTES levels were analyzed in the culture supernatants of cells from five donors treated with Lov, Mev, Sim(A) and Sim(N) on day 4 of treatment and compared to untreated control cells. (B) Mean levels of RANTES mRNA expression after treatment with the statins in four donors. The results are presented as a ratio between untreated (unt.) cells and cells treated (tr.) with statins.

Since we observed that statins could marginally down-regulate CCR5 expression at the cell surface of CD4^+^ T lymphocytes we wished to identify whether statin treatment had any effect on modulating RANTES production. We assayed the culture supernatants of CD4^+^ T lymphocytes treated either with or without statins for levels of RANTES production. From this we identified that for the statins tested there is a trend towards an increase in RANTES secretion (Figure 2A). When we compared mRNA expression of RANTES from the same cells untreated or treated with statins utilizing a specifically developed NASBA quantification assay we observed a small but insignificant increase in expression (Figure 2B). This increase in RANTES mRNA expression also suggests that the concentrations of statins used in the experiments are not toxic to the cells.

The results on CCR5 expression and RANTES secretion collectively suggest that statins have the effect of modulating chemokine and chemokine receptor expression levels. The lowering of CCR5 cell surface expression is associated with an increased production of RANTES, possibly reflecting a disruption to cell signalling or a reduced uptake and degradation of secreted RANTES by the CD4^+^ T lymphocyte from the culture supernatant. An increase in RANTES mRNA expression may also provide a further block to HIV-1 replication through higher levels of RANTES competing with virus binding to CCR5. Furthermore, our results would indicate that statins could be utilized as immunomodulatory agents with the capacity to alter induced immune responses. It has been shown that the Th-1 and Th-2 cytokine secretory balance can be altered by statin treatment with the net effect of modulating induced immune responses against viral antigens [15], [22]–[24]. This could have implications for modulating the type of cellular immune response mounted in HIV-1 infected patients, with a preferential skewing towards a strengthened Th1 response and heightened CTL activity.

### Statins preferentially reduce the infection of CD4^+^ T lymphocytes with an R5 versus X4 virus

**Figure 3:**
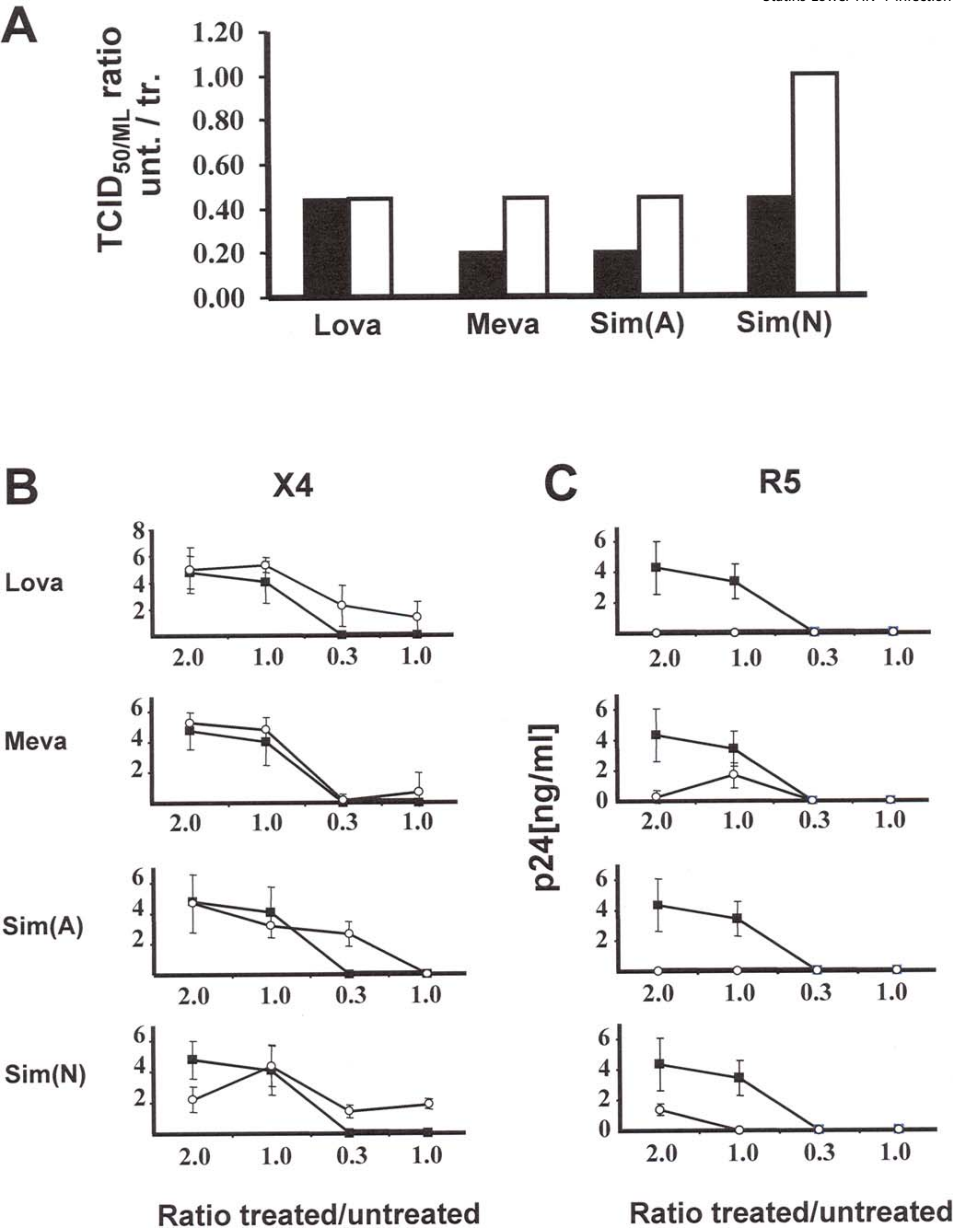
Stain treatment preferentially inhibits the replication of R5 versus X4 HIV-1 (A) TCID_50/ml_ infectious profiles of an R5 (black bars) and X4 virus (white bars) were monitored on CD4^+^ T lymphocytes in the presence of Lov, Mev, Sim(N) and Sim(A) and compared to non-statin treated cells. (B and C) CD4^+^ enriched lymphocytes were treated with Lov, Mev, Sim(A) and Sim(N) for 4 days then infected with an X4 (B) or R5 strain (C) of HIV-1 and cultured in the absence of statins as well as compared to non-statin treated cells. Non-statin treated cells are depict with closed squares and statin treated cells are depict with open circles.

Since the CCR5 CC-chemokine receptor and RANTES CC-chemokine expression levels have been modulated in CD4^+^ T lymphocytes we wished to identify whether HIV-1 infection could be modulated with statin treatment. Previous studies have shown that statins have the effect of lowering the in vitro replication of both R5 and X4 strains of HIV-1 equally [2]. We initially identified the overall effect of statins on reducing infection with an R5 and X4 virus on CD4^+^ T lymphocytes. For this purpose we performed 50% Tissue Culture Infectious Dose per ml (TCID_50/ml_) determinations of a X4 and an R5 virus in the continual presence of the four different statins and compared the results with non-statin treated cells (Figure 3A). The results demonstrate that both R5 and X4 viruses have a reduced TCID_50/ml_ when cultured in the presence of statins, with the R5 viruses being inhibited stronger with Mev, Sim(N) and Sim(A) than the X4 strain by approximately 2 fold, whilst inhibition with Lov was similar for both viruses.

We next identified whether pre-treatment of cells with statins could reduce infection with an R5 or X4 virus when culturing was performed free from statin treatment. We therefore treated CD4^+^ T lymphocytes with statins for 3 days before washing and infecting with an R5 or X4 strain of HIV-1. These were the same cultures where the CCR5 and RANTES expression levels had been determined above. After a short period of infection (12 hrs) the cells were washed and diluted onto fresh non-statin treated CD4^+^ cells and the resultant viral replication monitored. Pre-treatment of the cells with each statin separately had no effect on limiting infection with the X4 virus strain, whereas, statin pre-treatment had a major effect on limiting infection with an R5 variant at all cell concentrations tested (Figure 3B and 3C). These results demonstrate a clear distinction between infection with an R5 and X4 virus when the culturing is performed under statin free culture conditions.

This result indicate that statins can have a direct effect on limiting virus replication of both R5 and X4 viruses but also that R5 viruses may be preferentially inhibited further through a disruption in the CC-chemokine receptor and CC-chemokine expression patterns. This anti-viral effect would be in addition to the previously described mechanisms of statin inhibition induced through modulation of Rho GTPase activity as well as disruption to incorporation of LFA-1 into the virus membrane [3]. The preferential inhibition of R5 viruses and the disruption of CC-chemokine and CC-chemokine expression patterns that can modulate Th1, hence CTL responses, would advocate for the use of statins early in the infection history of a patient. Whether statins alone or in combination with other antiretroviral drugs could confer a clinical advantage needs to be addressed. The lowering of CCR5 cell surface expression has known clinical advantages since individuals heterozygous for the 32 bp deletion in their CCR5 gene are statistically more likely to harbor lower viral loads and progress slower in their disease course [25]. Interestingly, it has been shown that CD4^+^ T lymphocytes isolated from individuals heterozygous for the 32 bp deletion have lowered in vitro R5 infection profiles which correspond to lower CCR5 cell surface expression and increased RANTES production [26], [27]. The early intervention of HIV-1 infection with statins may therefore have the beneficial effect of providing a time-delay before having to prescribe the more conventional antiretroviral drug regimes. Alternatively, statins could be considered for prophylactic use in treating individuals at high risk of HIV-1 infection or those most recently exposed.

We demonstrate here that statin treatment of CD4^+^ T lymphocytes in vitro has the effect of reducing CCR5 cell surface expression and providing for the heightened secretion of RANTES in culture supernatant. Both CCR5 and RANTES mRNA expression levels show a trend towards a decrease and an increase, respectively. This altered regulation of chemokine and chemokine receptor expression correlates with the preferential reduction in infection of CD4^+^ T lymphocytes with an R5 versus X4 virus isolate. These results warrant for the further consideration of statins as anti-HIV-1 agents, especially in the early phases of infection or in a prophylactic capacity. Additionally, statins could be considered for use as immunomodulatory compounds such as in heightening or broadening immune responses during vaccination protocols.

## Material and Methods

### Statins

The following statins were utilized in this study: lovastatin, mevastatin and simvastatin. The latter was used both in active and non-active forms, meaning the activity to inhibit HMG-CoA reductase. Activation occurred by hydroxylation of the statin using NaOH applying standard protocols. The non-active form nevertheless can get active as a result of normal intracellular metabolic processes.

### Cells and statin treatment

Peripheral Blood Mononuclear Cells (PBMC) were isolated from human donors and treated with PHA (2 µg/ml) and recombinant IL-2 (100 units/ml) as described earlier [28]. The following day the activated PBMCs underwent CD8^+^ lymphocyte depletion according to the manufacturers instructions and the enriched CD4^+^ T lymphocytes were treated with filter sterilized stock solutions of various statins (5 mM), reaching final concentrations of 5 µM. Following incubation with statins for 3 days the cells were utilized in the various assays described below. Supernatants from the cells after 3 days of incubation with statins were harvested as after 20 hrs of culturing in the presence of the viruses and stored at −80°C.

### Viruses

We utilized virus SF162, a CCR5 using virus and an envelope modified LAI virus which had been altered in the V1V2 and V3 region as previously described (+6X.10ΔgV3) and that was of the X4 phenotype [28].

### Flow cytometry

The cells were stained using anti-CD4-PerCP, anti-CCR5-FITC, anti-CD4- PE (all from BD PharMingen) using standard labelling protocols. Signals were acquired on a FACS Calibur flow cytometer (BD Biosciences) with CellQuest software (BD Biosciences).

### Enzyme immunoassay

Levels of RANTES in cell supernatants were measured with specific ELISA kits from R&D Systems according to the manufacturer's instructions.

### HIV infection assay

Viruses were monitored for their TCID_50/ml_ values in the absence and presence of statins (all at 5 mM final concentration). Briefly, PHA activated CD4^+^-enriched lymphocytes (as obtained above) were plated at 2×10^5^ cells/well in 96-well plates with 5 fold serial dilutions of the virus (8 replicas). On day 7 the medium was replenished and on day 14 the p24 levels were determined utilizing a standard p24 ELISA assay, with the TCID_50/ml_ calculated. Virus replication assays were performed on statin treated or untreated CD4^+^ T lymphocytes. Cells were washed with PBS, resuspended in RPMI culture medium (containing 10% FCS and pen/strep) at a concentration of 4×10^6^ cells/ml in the absence or presence of statins (5 µM). On day 4 of culture the cells were washed, counted and then incubated with HIV-1 viral stocks at 100 TCID_50_ per well in 96 well culture plates. The virus was incubated for 12 hrs with the cells in 200 µl (2×10^5^ cells/well), washed twice with PBS, resuspended in 200 µl of RPMI (containing 10% FCS and pen/strep) and cultured at 37^o^C for 10 days with all infections performed in triplicate. The infected cells were co-cultured in different ratios of infected to non-infected cells (fixed amount at 1×10^5^ cells). Cultures were fed with fresh media on days 4 and 7 and HIV CA-p24 was determined in culture supernatant on day 10 using a standard CA-p24 ELISA assay.

### NASBA

RNA was isolated from 3×10^5^ cells using a silica-based total nucleic acid isolation protocol. One tenth of the total nucleic acids were used for three different reactions to quantify the number of nuclear genomic molecules, the number of CCR5 mRNA molecules and the number of RANTES mRNA molecules. The NASBA reactions contained the following reagents: Tris-HCl (40 mM, pH = 8.3), MgCl_2_ (12 mM), KCl (90 mM), DTT (5 mM), dNTPs (1 mM each), rNTPs (2 mM each), DMSO (15% v/v), oligonucleotides: target-P1 and target-P2 (0.2 µM each) and the target molecular beacons (0.04 µM each) to detect the amplicons in real-time (Table 1). If the number of genomic DNA copies was determined, the samples were incubated for 12 minutes at 37°C with 2 units MspI per reaction to allow the restriction enzyme digestion, subsequently heated to 95°C for 3 min. to inactivate the MspI enzyme and to denature the DNA allowing efficient annealing of the P1-primers after cooling to 41°C. For the mRNA assays, the samples were incubated 5 min. at 65°C, before cooling to 41°C. After 1 min. at 41°C, the NASBA enzyme mixtures were added (BSA [2.1 mg], RNase H [0.08 units], T7 RNA polymerase [64 units], AMV-RT [9.6 units] and sorbitol [0.375 M] per reaction) after which the amplification was followed for 60 min. at 41°C in a fluorescence reader with a thermostat, measuring every 30 sec. the fluorescein (extinction: 485 nm/emission: 518 nm) signals. All samples were assayed in duplicate.

**Table 1:** Probes and primers for CCR5 and RANTES NASBA assay's

**CCR5**	
Primer 5′ (5′→3′):	AAT TCT AAT ACG ACT CAC TAT AGG GAG CAG CGG CAG GAC CAG CCC CA
Primer 3′ (5′→3′):	TTT GGG GTG GTG ACA AGT GTG ATC A
Probe (5′→3′):	FAM – CGT ACG TCC ATA CAG TCA GTA TCA ATT CGT ACG
**RANTES**	
Primer 5′ (5′→3′):	AAT TCT AAT ACG ACT CAC TAT AGG GAA ACA GGC AAA TTT GTG TAA GTT CA
Primer 3′ (5′→3′):	TGC CCC GTG CCC ACA TCA AGG A
Probe (5′→3′):	FAM – GCA TGC TCA CCC GAA AGA AGC GCC AAG CAT GC

### Statistical analyses

Students T-test in the case of normal distributed data was used to analyse significant differences between sets of data. Non-parametric tests as the Wilcoxon Rank test were used to analyse significance between data sets in case of not-normally distributed data. Data are presented as mean+/−SD with P values <0.05 (two-sided) considered as statistically significant.
